# Non-invasive detection of hTERT mRNA in deep-cough swabs for early diagnosis of lung cancer

**DOI:** 10.3389/fmolb.2026.1798729

**Published:** 2026-03-05

**Authors:** Yinhua Liu, Xi Huang, Bing Zhou, Wu Sun, Yu He, Shengnan Xu, Junqiang Lu, Ran Chen, Guoliang Mao, Guoping Zhu

**Affiliations:** 1 Anhui Provincial Engineering Research Centre for Molecular Detection and Diagnostics, College of Life Sciences, Anhui Normal University, Wuhu, China; 2 Department of Pathology, Wannan Medical College First Affiliated Hospital, Yijishan Hospital, Wuhu, China; 3 Anhui Provincial Key Laboratory of Molecular Enzymology and Mechanism of Major Diseases, College of Life Sciences, Anhui Normal University, Wuhu, China; 4 Zhejiang JFK Biological Technology Inc Ltd., Hangzhou, China

**Keywords:** deep-cough swab, early diagnosis, exosomes, hTERT mRNA, lung cancer, non-invasive biomarker, RT-qPCR

## Abstract

**Introduction:**

Lung cancer remains the leading cause of cancer-related mortality globally, with current screening methods like low-dose computed tomography (LDCT) limited by radiation risks and false negatives. Non-invasive diagnostic tools are urgently needed.

**Methods:**

We developed a deep-cough swab protocol coupled with rapid RNA extraction and direct RT-qPCR to detect human telomerase reverse transcriptase (hTERT) mRNA in airway secretions. The assay was validated using exosomes from lung cancer cell lines and applied to 300 participants (106 early-stage lung cancer patients, 142 benign pulmonary nodule cases, and 52 healthy controls).

**Results:**

The assay achieved 90.6% sensitivity (95% CI: 85.1%–96.2%) for stage IA lung cancer and 95.9% specificity (95% CI: 93.1%–98.7%) in non-cancer controls. ROC analysis demonstrated excellent discrimination (AUC = 0.97, p < 0.0001). Sensitivity was consistent across histological subtypes and unaffected by smoking status (p > 0.05).

**Conclusion:**

Deep-cough swab detection of hTERT mRNA provides a clinically viable, non-invasive method for auxiliary diagnosis of early-stage lung cancer, potentially complementing CT screening for pulmonary nodule management.

## 1 Introduction

Lung cancer has maintained the highest mortality rate among cancers globally for over a decade, with approximately 2.5 million new cases and 1.8 million deaths reported in 2022 alone (Guo et al., 2024; Zhou et al., 2024; Ji et al., 2025). Risk factors are increasingly diverse, encompassing smoking (including secondhand smoke) and Chronic Obstructive Pulmonary Disease (COPD), PM2.5, occupational exposures, genetic susceptibility (e.g., 35% EGFR mutation prevalence), and COVID-19 infection, collectively posing significant challenges for prevention and control (Li et al., 2015; Chen et al., 2020; Conibear et al., 2022). While low-dose computed tomography (LDCT) screening has become the standard for detection, it faces significant limitations including lengthy follow-up intervals, radiation-induced carcinogenicity concerns, high false-negative rates for central tumors, substantial overdiagnosis and widespread anxiety (Li et al., 2017; Rampinelli et al., 2017; Li et al., 2020; Wang et al., 2023; Smith-Bindman et al., 2025). These challenges are particularly pronounced in China, where lung cancer epidemiology shows distinct characteristics including rapid incidence growth and low early diagnosis rates (Han et al., 2024).

The detection of molecular biomarkers in biological fluids offers a promising complementary approach to imaging-based screening. Human telomerase reverse transcriptase (hTERT) mRNA represents an ideal candidate biomarker, as its expression is activated in over 95% of malignant tissues while remaining silent in normal lung cells (Chen et al., 2003; Fujita et al., 2003). Reactivation of hTERT occurs early in carcinogenesis, providing a potential window for early detection (Akincilar et al., 2016; Leao et al., 2018; Dratwa, et al., 2020; Tornesello et al., 2023; Liu, et al., 2024). Furthermore, cancer cells actively secrete hTERT mRNA via exosomes, which protect the RNA from degradation in airway secretions (Batagov and Kurochkin, 2013; Gutkin et al., 2016; Goldvaser et al., 2017).

While previous studies have demonstrated the feasibility of detecting hTERT mRNA in sputum samples, practical limitations including inconsistent sample quality and difficulty obtaining adequate specimens from early-stage patients have hindered clinical application (Chen et al., 2019). To overcome these limitations, we developed a standardized deep-cough swab sampling method that reliably collects airway mucus from the oropharynx. This approach, combined with optimized RNA extraction and direct RT-qPCR detection, provides a robust platform for non-invasive lung cancer detection.

## 2 Materials and methods

### 2.1 Ethics statement

The study was approved by the Ethics Committee of Yijishan Hospital Affiliated to Wannan Medical College (No. 2024-195) and registered at the Chinese Clinical Trial Registry. All participants provided written informed consent.

### 2.2 Study population and design

A total of 300 participants were enrolled between January and July 2025, including 106 patients with pathologically confirmed early-stage lung cancer (stage IA1-IA3), 142 patients with benign pulmonary nodules, and 52 healthy controls. Inclusion criteria required age 30–85 years and possession of at least one of the following risk factors: smoking history (including secondhand smoke exposure), chronic obstructive pulmonary disease (COPD), PM2.5 exposure, occupational exposure (e.g., air pollution, radiation), EGFR mutation (a known lung cancer susceptibility gene), or history of COVID-19 infection. Exclusion criteria included prior cancer diagnosis, severe cardiopulmonary disease, or inability to provide informed consent.

### 2.3 Deep-cough swab sampling protocol

Participants performed a standardized sampling procedure: (1) drinking water to hydrate the oral cavity, (2) percussing the anterior chest 10 times, (3) performing 6 deep coughs, (4) gentle expectoration to remove excess oral fluid, and (5) oropharyngeal swabbing of the posterior pharynx for 5–10 s using sterile polyester-tipped swabs. Swabs were immediately immersed in storage buffer (5 M guanidine hydrochloride, 0.2% DEPC, 10 μg/mL diatomaceous earth) and transported at ambient temperature.

### 2.4 RNA extraction and RT-qPCR

RNA Extraction and RT-qPCR: A divalent silica-based rapid RNA extraction method was used. Briefly, after centrifugation of the swab eluate and washing with an acetate-ethanol buffer, RNA was eluted in a reverse transcription-ready buffer containing target-specific primers. Direct RT-qPCR was performed using SYBR Green detection with the following cycling conditions: 65 °C for 15 min; 40 cycles of 94 °C for 5 s, 65 °C for 35 s. Result interpretation: Ct < 33: positive (successful amplification); Ct ≥ 33 or no Ct value: negative (no amplification). Primer sequences: MUC5AC-F: CGTACCAGAACCAGTCGACCTG, MUC5AC-R: CCTCGAGCGAGTACATGGAAGAG; hTERT-F: GCCTTCAAGAGCCACGTCTCTAC, hTERT-R: GGGAGGAGCTCTGCTCGATG. Primer concentration: 0.5 μM; reaction volume: 30 μL; RNA elution volume: 30 μL. SYBR Green Master Mix: Thermo Fisher Scientific, Cat. No. 4367659; Primers order: Shanghai Sangon.

### 2.5 Cell culture and exosome preparation

Exosomes were precipitated from conditioned media using PEG/NaCl and characterized for hTERT mRNA expression to establish assay sensitivity. Briefly, lung cancer cell lines (A549, Calu-1, NCI-H1299, NCI-H446) were cultured in RPMI-1640 with 10% FBS and 1% Penicillin-Streptomycin (P/S). Cultures were maintained without medium change for 2–4 days until confluent. 30 mL of culture supernatant was collected into 50 mL centrifuge tubes and centrifuged at 4 °C, 3,000 rpm for 5 min 27 mL of the supernatant was carefully transferred to a new 50 mL tube pre-filled with 3 mL of precipitation aid (1.5 M NaCl, 16% w/v PEG6000, 0.5% v/v DEPC). This mixture was centrifuged at 4 °C, 12,000 rpm for 30 min. The supernatant was carefully aspirated, and the pellet was resuspended in 3 mL of storage buffer (1× PBS, 0.2% v/v DEPC, 50% v/v glycerol). The resuspended exosomes were aliquoted (150 μL per tube) into 2 mL cryovials and stored at −80 °C. All reagents were purchased from Thermo Fisher Scientific (for cell cultures) or Sigma (for exosome preparation).

### 2.6 Statistical analysis

Diagnostic performance was assessed using receiver operating characteristic (ROC) curve analysis. Sensitivity, specificity, positive and negative predictive values were calculated with 95% confidence intervals. Group comparisons used Fisher’s exact test or McNemar test as appropriate, with p < 0.05 considered significant.

## 3 Results

### 3.1 Assay development and validation

**FIGURE 1:**
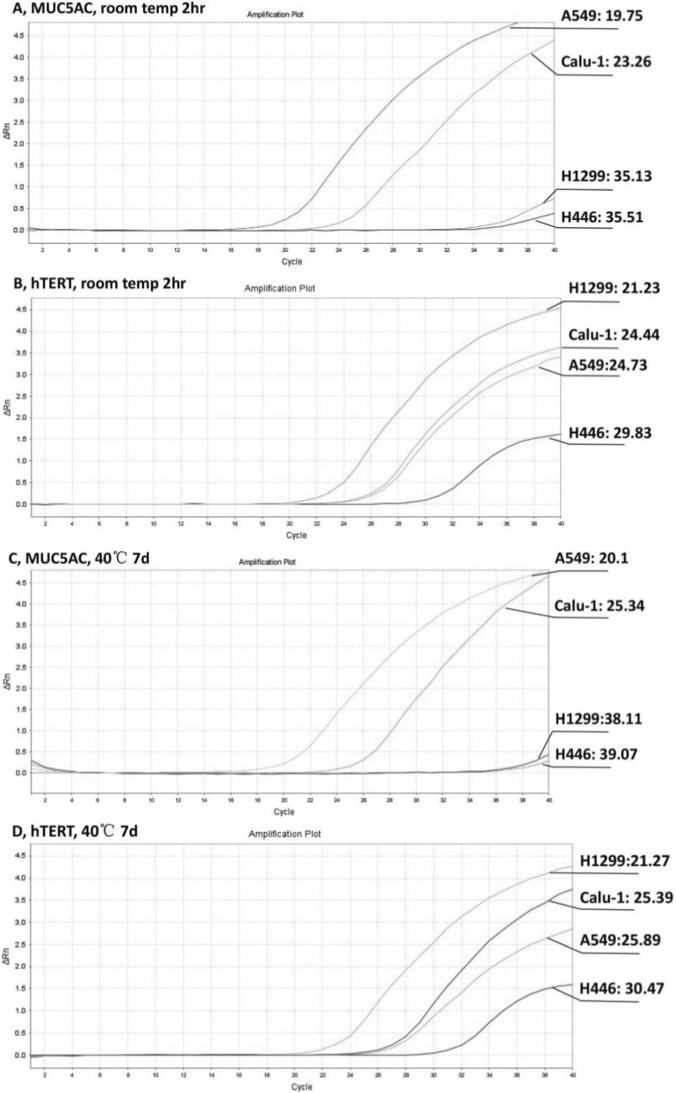
The representative RT-qPCR amplification plots of exosomal MUC5AC and hTERT mRNAs from 4 lines of lung cancer cells. **(A,C)** MUC5AC mRNA amplification plots for samples tested within 2 h **(A)** and after storage at 40 °C for 7 days **(C)**. **(B,D)** hTERT mRNA amplification plots for samples tested within 2 h **(B)** and after storage at 40 °C for 7 days **(D)**. Each curve was labeled with the line symbol and the Ct value, e.g., A549:19.75.

After immersion and stirring of thawed exosome samples, consistency in the adsorbed sample volume was confirmed by weighing swabs before/after sampling and measuring residual volume (100 ± 10 μL). Stability testing compared samples tested within 2 h of sampling (Groups A, B, n = 20 each, triplicate runs) versus samples stored at 40 °C for 7 days before testing (Groups C, D, n = 20 each, triplicate runs). Amplification curves for MUC5AC mRNA (Figures 1A,C) and hTERT mRNA (Figures 1B,D) remained similar under both conditions, indicating that the storage buffer preserved swab samples stably at 40 °C for 7 days. Analysis of Ct values revealed strong MUC5AC mRNA expression in A549 exosomes (Ct < 20), significant expression in Calu-1 exosomes (Ct < 33), and no/low expression in NCI-H1299 and NCI-H446 exosomes (Ct > 35). All four cell lines exhibited significant hTERT mRNA expression (Ct < 33), with particularly strong signals in A549 and NCI-H1299 (Ct < 25).

### 3.2 Comparison of volunteer swab sampling methods

**FIGURE 2:**
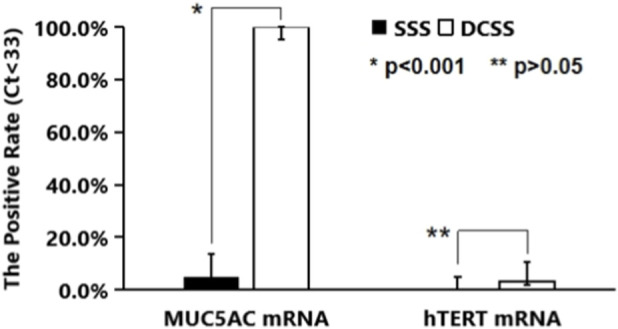
The positive rates of Saliva-Swab-Sampling (SSS) and Deep-Cough-Swab-Sampling (DCSS) RT-qPCR tests of MUC5AC and hTERT mRNAs from healthy volunteers.

MUC5AC mRNA was used as a marker for airway mucus because MUC5AC is a major component of airway mucus, and its expression is directly related to airway mucus secretion (Chen et al., 2004). Using the established RT-qPCR assays (positive defined as Ct < 33), direct oral swab sampling (saliva swab) yielded a MUC5AC mRNA detection rate of 5.0% (3/60, 95% CI: 2.1%–13.7%) and an hTERT mRNA detection rate of 0% (0/60, 95% CI: 0.0%–5.1%). Deep-cough swab sampling (drink water - percuss chest 10 times - perform 6 deep coughs - expectorate excess fluid - swab posterior oropharynx) yielded a MUC5AC mRNA detection rate of 100% (60/60, 95% CI: 95.1%–100%) and an hTERT mRNA detection rate of 3.3% (2/60, 95% CI: 1.9%–10.4%) (Figure 2). Fisher’s exact test revealed a highly significant difference in MUC5AC mRNA detection rates (p < 0.001), confirming that the deep-cough swab process effectively samples airway mucus, consistent with MUC5AC being an airway mucus biomarker (Chen et al., 2004). The difference in hTERT mRNA detection rates was not significant (p > 0.05); larger studies are needed to establish reliable conclusions in healthy volunteers. Both volunteers testing positive for hTERT mRNA underwent annual LDCT, revealing 3–5 mm ground-glass nodules; they were advised on nutrition/rest and scheduled for follow-up LDCT.

### 3.3 Characteristics of the clinical study cohort

**FIGURE 3:**
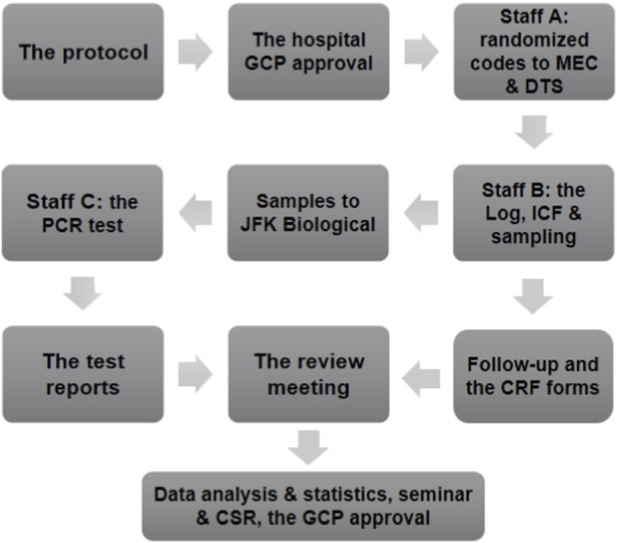
Study flow chart.

The study followed the approved protocol (Figure 3). Between January 2025 and July 2025, 300 participants were enrolled from Yijishan Hospital (Health Center and Thoracic Surgery). All samples were collected before definitive health/diagnostic results were available. Final groups based on follow-up/diagnosis were: Non-malignant Controls (n = 194): Included 52/194 (26.8%) healthy individuals (no symptoms, no pulmonary nodules confirmed by health exam/LDCT) and 142/194 (73.2%) with benign pulmonary nodules (BPN: 84/142 (59.2%) <6 mm, 39/142 (27.5%) 6–14 mm, 19/142 (13.4%) ≥15 mm). Smoking history: 48/106 (45.3%) lung cancer patients; 75/194 (38.7%) controls. Occupational exposures: 21/106 (19.8%) lung cancer patients; 22/194 (11.3%) controls. Chronic cough: 16/106 (15.1%) lung cancer patients; 14/194 (7.2%) controls. Early-Stage Lung Cancer (n = 106): Stages IA1 (n = 56), IA2 (n = 32), IA3 (n = 18). Types: Adenocarcinoma (LUAD, n = 90), Squamous Cell Carcinoma (SCC, n = 11), Small Cell Lung Cancer (SCLC, n = 5). Detailed cohort characteristics are summarized in Table 1.

**TABLE 1:** Clinicopathological characteristics of participants.

Characteristics	Parameters	Healthy	BPN	Lung cancer
Ages	30–49	36 (69.2%)	51 (35.9%)	33 (31.1%)
	50–85	16 (30.8%)	91 (64.1%)	73 (68.9%)
Sex	Male	27 (51.9%)	74 (52.1%)	65 (61.3%)
	Female	25 (48.1%)	68 (47.9%)	41 (38.7%)
Smoking (family)	N	35 (67.3%)	84 (59.2%)	58 (54.7%)
	Y	17 (32.7%)	58 (40.8%)	48 (45.3%)
Occupational exposures	N	50 (96.2%)	122 (85.9%)	85 (80.2%)
	Y	2 (0.8%)	20 (14.1%)	21 (19.8%)
Chronic cough	N	52 (100%)	128 (90.1%)	90 (84.9%)
	Y	0 (0%)	14 (9.9%)	16 (15.1%)
Pulmonary nodule	N	-	-	-
	<6 mm	-	84 (59.2%)	0
	6–14 mm	-	39 (27.5%)	64 (60.4%)
	≥15 mm	-	19 (13.4%)	42 (39.6%)
Lung cancer	N	-	-	-
	Stage IA1	-	-	56 (52.8%)
	IA2	-	-	32 (30.2%)
	IA3	-	-	18 (17.0%)
	LUAD	-	-	90 (84.9%)
	SCC			
	SCLC	-	-	16 (15.1%)

BPN, benign pulmonary nodule.

### 3.4 ROC curve analysis

**FIGURE 4:**
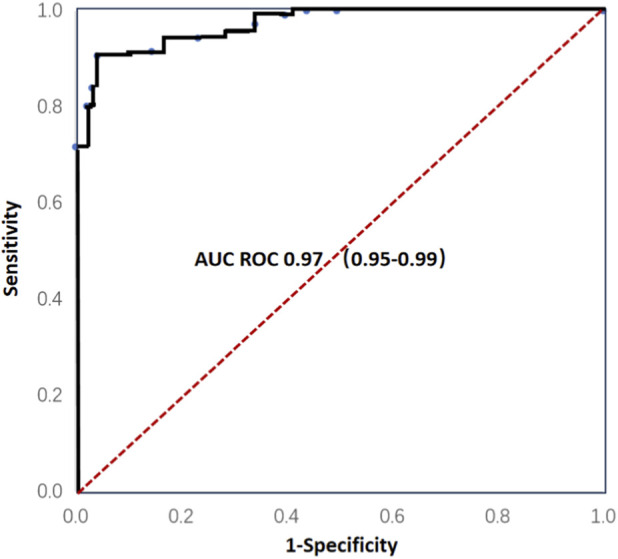
ROC curve of hTERT mRNA showing discrimination between non-malignancy and early-stage lung cancers.

We evaluated the expression of hTERT mRNA in the swab samples from 106 patients with early stage lung cancer and 194 non-cancer donors that includes 142 patients with non-cancer pulmonary nodules and 52 healthy individuals. We observed significant amplification from the samples of patients with lung cancer while no significant amplification in donors without lung cancer. Ct values that is inversely proportional to the level of hTERT mRNA both for patients with lung cancer and non-cancer group were plotted into ROC curve to assess cutoff values for distinguishing patients with lung cancer from donors without lung cancer (Table 2). The ROC analysis demonstrated exceptional diagnostic accuracy (AUC = 0.97, 95% CI 0.95–0.99, p < 0.0001) at Ct = 33 cutoff (Figure 4). When the Ct value is smaller than 33, the test result would be defined as positive whereas the Ct value was equal to or larger than 33 or showing “No Ct”, the result would be defined as negative, resulting in specificity of 95.9% and sensitivity of 90.6% for distinguishing patients with lung cancer (early stages) from non-malignant controls, reflecting hTERT’s near-universal expression in malignancies.

**TABLE 2:** Sensitivity and specificity of hTERT mRNA (Ct values) in discriminating between early-stage lung cancer and non-malignant controls.

Cut-off (Ct value)	Sensitivity	95% CI	Specificity	95% CI
No Ct	100% (106/106)	-	0%	-
39	100% (106/106)	-	50.5% (98/194)	43.5%–57.6%
38	100% (106/106)	-	56.2% (109/194)	49.4%–62.9%
37	99.1% (105/106)	97.6%–100%	60.3% (117/194)	53.7%–66.9%
36	97.2% (103/106)	93.7%–98.9%	66.0% (128/194)	59.6%–72.4%
35	94.3% (100/106)	91.3%–96.8%	76.8% (149/194)	70.9%–82.7%
34	91.5% (97/106)	88.4%–94.3%	85.6% (166/194)	80.9%–90.2%
**33**	**90.6% (96/106)**	**85.1%-96.2%**	**95.9% (186/194)**	**92.9%-98.9%**
32	84.0% (89/106)	77.3%–90.6%	96.9% (188/194)	93.9%–99.9%
31	80.2% (85/106)	71.8%–86.7%	97.9% (190/194)	95.2%–100%
30	71.7% (76/106)	63.1%–80.3%	100% (194/194)	-

Bold values indicate the optimal cut-off (Ct < 33) selected based on the highest Youden Index for distinguishing early-stage lung cancer from non-malignant controls.

### 3.5 Stratified analysis by clinical parameters

Sensitivity: Among 106 lung cancer patients, 90.6% (96/106, 95% CI: 85.1%–96.2%) were hTERT mRNA-positive. Specificity: Among 194 non-malignant controls, 95.9% (186/194, 95% CI: 93.1%–98.7%) were hTERT mRNA-negative. Accuracy (Total Agreement): 94.0% (95% CI: 91.3%–96.7%). Positive Predictive Value (PPV): 92.3%. Negative Predictive Value (NPV): 94.9%. Youden Index (YI): 86.5%. Cohen’s Kappa value was 0.87 > 0.75 (P < 0.01), indicating excellent agreement between hTERT mRNA tests and clinicopathological diagnosis (Table 3).

**TABLE 3:** Diagnostic characteristics of hTERT mRNA: Sensitivity, Specificity, PPV (Positive Predictive Value), NPV (Negative Predictive Value), YI (Youden index) and Kappa value.

Marker	Sensitivity	Specificity	Accuracy	PPV	NPV	YI	Kappa
hTERT mRNA	90.6%	95.9%	94.0%	92.3%	94.9%	86.5%	0.87

**FIGURE 5:**
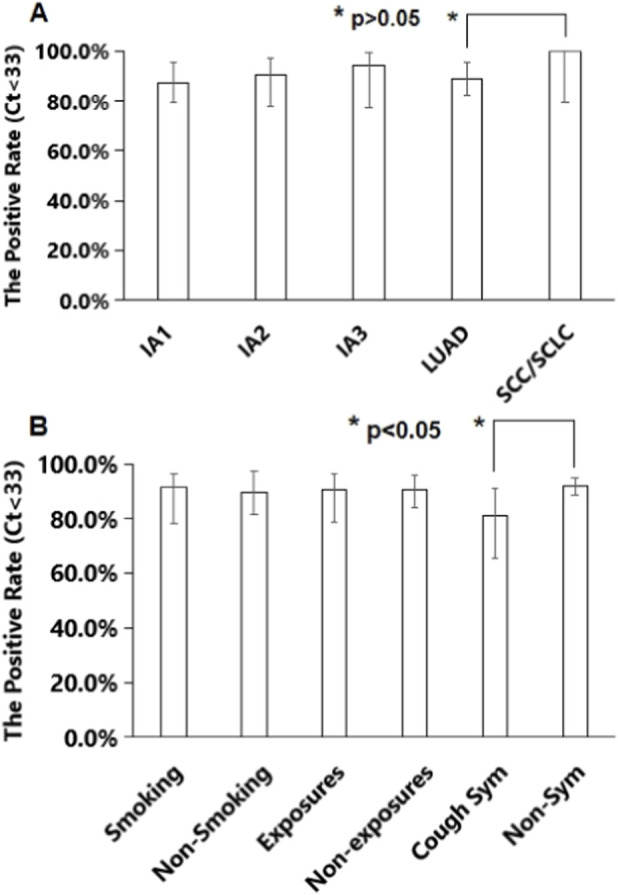
The positive rates of hTERT mRNA tests of lung cancer samples. **(A)** Stages and types; **(B)** High-risk factors. Sym, symptoms.

Stratification by Cancer Stage: hTERT mRNA was detected in 87.5% (49/56, 95% CI: 79.3%–95.6%) of stage IA1, 90.6% (29/32, 95% CI: 78.0%–97.0%) of stage IA2, and 94.4% (17/18, 95% CI: 77.3%–99.3%) of stage IA3 samples, demonstrating high sensitivity across stage I lung cancers, suggesting potential for early molecular diagnosis (Figure 5A).

Stratification by Histological Subtype: Among 90 LUAD cases, 88.9% (80/90, 95% CI: 82.4%–95.4%) were hTERT mRNA-positive. All 16 non-LUAD cases (11 SCC, 5 SCLC) were positive (100%, 95% CI: 79.5%–100%). Sensitivity in non-LUAD cases was insignificantly higher than in LUAD cases (p > 0.05), suggesting the test is not subtype-dependent (Figure 5A).

Association with Risk Factors/Symptoms: Smoking (incl. passive): Sensitivity was 89.7% (52/58, 95% CI: 81.8%–97.5%) in non-smokers and 91.7% (44/48, 95% CI: 78.3%–96.2%) in smokers (p > 0.05). Occupational Exposure (e.g., air pollution, radiation): Sensitivity was 90.6% (77/85, 95% CI: 84.3%–96.1%) in non-exposed and 90.5% (19/21, 95% CI: 78.7%–96.5%) in exposed (p > 0.05). Symptoms: Sensitivity was 92.2% (83/90, 95% CI: 88.5%–95.0%) in asymptomatic patients and 81.3% (13/16, 95% CI: 65.6%–91.2%) in patients with chronic cough (p < 0.05) (Figure 5B).

### 3.6 Specificity in control populations

**FIGURE 6:**
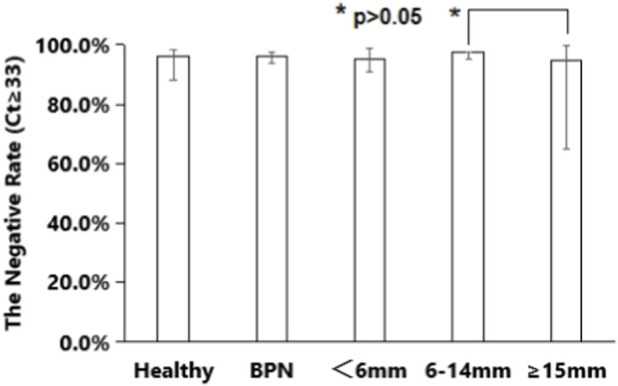
The negative rates of hTERT mRNA tests of non-malignancy samples. BPN, benign pulmonary nodules.

The assay maintained high specificity across control groups: Healthy Donors: 96.2% (50/52, 95% CI: 88.3%–98.1%) tested negative. Benign Pulmonary Nodules (BPN): Overall, 95.8% (136/142, 95% CI: 93.8%–97.2%) tested negative. By nodule size: <6 mm: 95.2% (80/84, 95% CI: 90.7%–99.8%); 6–14 mm: 97.4% (38/39, 95% CI: 94.9%–97.6%); ≥15 mm: 94.7% (18/19, 95% CI: 64.7%–99.9%) (p > 0.05 between size subgroups) (Figure 6).

Among the 194 non-malignant controls, 8 cases (4.1%) were hTERT mRNA positive (false positives). The clinical characteristics of these false-positive cases included: 6 cases with <6 mm pulmonary nodules and 2 cases with 6–14 mm pulmonary nodules; 3 cases had a history of chronic cough, 2 had a smoking history, and 3 had a history of occupational exposure. None of the 8 patients developed lung cancer during a 1-year follow-up period, but pulmonary nodules increased in size (from <6 mm to 6–14 mm) in 4 cases during follow-up. We speculate that these false positives may be related to increased hTERT mRNA expression due to airway inflammation or infection, rather than lung cancer.

### 3.7 Discussion

This study establishes deep-cough swab sampling coupled with hTERT mRNA detection as a robust method for non-invasive lung cancer detection. The approach addresses critical limitations of current screening methods by providing a safe, radiation-free alternative with high sensitivity for early-stage disease. Several aspects of our findings warrant particular emphasis.

The 90.6% sensitivity for stage IA cancers represents a significant advancement over existing molecular approaches, particularly given the challenges of detecting early-stage disease. This performance exceeds reported sensitivities for DNA methylation tests, which typically range from 70% to 85% for early-stage lesions (Weiss et al., 2017; Li et al., 2022). The superior sensitivity likely reflects both the biological characteristics of hTERT expression and technical advantages of our detection method. Circulating tumor DNA (ctDNA) or shed cell DNA concentrations in blood or sputum from early-stage lung cancer patients are usually below the detection limits of DNA methylation tests while the mandatory bisulfite conversion step causes substantial DNA fragmentation and template loss, further compromising sensitivity. Besides, sputum sampling based DNA methylation tests face not only the challenges of sample collection and quality control mentioned earlier, but also significant difficulties in nucleic acid extraction from sputum specimens: the process requires the use of toxic and irritant organic solvents such as phenol and chloroform, which are cumbersome to handle and prone to interference from pH levels and salt concentrations in the system, resulting in inconsistency of results (Xu et al., 2019). As a central regulator of cellular immortality, hTERT activation occurs early in transformation and is maintained throughout progression (Leao et al., 2018). Furthermore, the exosome-mediated secretion mechanism enables accumulation of hTERT mRNA in airway secretions independent of cell exfoliation, potentially allowing detection before morphological changes become apparent on CT.

We noted that the sensitivity of hTERT mRNA detection was lower in chronic cough patients (81.3%) compared to asymptomatic patients (92.2%), a finding that appears counterintuitive. We propose that repeated coughing may lead to the depletion of hTERT mRNA in airway mucus, reducing detection sensitivity. We suggest implementing a modified sampling protocol for chronic cough patients, such as recommending self-sampling early in the morning to avoid excessive loss of hTERT mRNA due to frequent coughing throughout the day.

The high specificity (95.9%) is equally noteworthy, as false positives represent a major concern in cancer screening. The limited expression of hTERT in benign conditions contrasts with methylation markers, which can show field effects in histologically normal tissue exposed to carcinogens (Weiss et al., 2017). This biological specificity, combined with the optimized Ct cutoff, minimizes unnecessary follow-up procedures while maintaining sensitivity. The performance of this test was unaffected by smoking history (no significant difference in sensitivity between smokers and non-smokers, p = 0.82) or pathological subtype (no significant difference in sensitivity between adenocarcinoma and non-adenocarcinoma, p = 0.096), indicating its suitability for broad-based screening. The analysis of false-positive cases suggests that hTERT mRNA may be expressed not only in lung cancer but also in some benign pulmonary diseases. This emphasizes the need for comprehensive evaluation combining imaging examinations (such as LDCT) in clinical applications to avoid over-diagnosis. Future studies should further explore the pathological mechanisms underlying these false-positive cases to optimize the detection threshold.

Technical innovations contribute significantly to the assay’s performance. The deep-cough swab protocol ensures consistent sampling of lower airway secretions, as demonstrated by 100% detection rate of the mucus biomarker MUC5AC mRNA, effectively addressing the challenge of sample acquisition in early-stage lung cancer patients and individuals with pulmonary nodules who often lack productive cough symptoms. The sampling method also showed excellent stability, with consistent hTERT mRNA detection from exosome samples stored for 7 days at 40 °C. The stability of samples in storage buffer facilitates transportation without cold chain requirements, enhancing practicality for screening programs. The integrated RNA extraction and RT-qPCR method preserves RNA integrity while simplifying processing, making the assay suitable for clinical implementation.

This study did not employ internal reference genes for RNA quantification correction, as we utilized a detection method that qualitatively assesses expression levels based on the amplification Ct value of hTERT mRNA. The sensitivity and specificity of this method were determined by ROC analysis (AUC = 0.97, 95% CI: 0.95–0.99, p < 0.0001), with a Ct value <33 established as the positive threshold. We selected this approach to streamline the detection process, improve efficiency, and enhance its suitability for large-scale screening applications.

As a potential screening tool, this assay demonstrates significant cost-effectiveness advantages. The estimated cost per test is $10–$20, lower than LDCT ($30–$50 per scan). The sampling process is non-invasive and self-administered, and the detection process takes only 1.5 h, whereas LDCT requires appointment scheduling, scanning, and radiological analysis, typically taking 1 day. This method requires only standard RT-qPCR equipment, available in most hospital laboratories, with no need for specialized equipment. Based on our preliminary estimates, screening individuals over 50 years old with this test could significantly reduce professional labor expenditure. For example, screening 5,000 individuals could save approximately $136,500. Future population screening studies and more comprehensive health economic evaluations, including long-term cost-effectiveness analyses, should be conducted.

### 3.8 Limitations and future directions

Several limitations should be considered. The study included relatively few cases of central tumors (squamous cell and small cell carcinomas), requiring validation in larger cohorts. Long-term follow-up data are needed to assess the prognostic significance of hTERT mRNA detection. Future studies should compare the assay directly with LDCT and other molecular methods in prospective screening populations. The temperature stability study used exosomes from lung cancer cell lines, not clinical samples. We acknowledge that the stability of clinical samples at 40 °C for 7 days has not been validated, and patient-derived exosomes may differ in purity and composition. Therefore, we recommend more comprehensive stability testing of clinical samples before clinical application.

Technical enhancements could further improve performance. Incorporating additional biomarkers such as cancer-associated miRNAs or mitochondrial PRODH may increase sensitivity for cancer detection (Powrozek et al., 2015; Zhao et al., 2022; Xi et al., 2024). Automation of the sampling and testing process would enhance reproducibility and scalability for population-level screening.

## 4 Conclusion

Deep-cough swab detection of hTERT mRNA represents a significant advance in non-invasive lung cancer diagnosis. The method combines high sensitivity for early-stage disease with practical advantages including simplicity, safety, and cost-effectiveness. As an auxiliary tool complementing CT screening, it addresses critical limitations of current approaches and shows potential for improving early detection rates. Further validation in diverse populations and settings will establish its role in lung cancer control strategies.

## Data Availability

The datasets presented in this study can be found in online repositories. The names of the repository/repositories and accession number(s) can be found in the article/supplementary material.
